# 1-[(2-Methyl­piperidin-1-yl)(phen­yl)meth­yl]naphthalen-2-ol

**DOI:** 10.1107/S1600536812038652

**Published:** 2012-09-15

**Authors:** Yao Huang

**Affiliations:** a School of Chemical Engineering, Yunnan Radio and TV University, Kunming 650023, People’s Republic of China

## Abstract

In the title compound, C_23_H_25_NO, an intra­molecular O—H⋯N hydrogen bond defines the mol­ecular conformation; the naphthol mean plane and the benzene ring form a dihedral angle of 75.8 (2)°. The piperidine ring adopts a chair conformation. The crystal packing exhibits no short inter­molecular contacts.

## Related literature

For the crystal structures of related compounds, see: Wang & Zhao (2009 ▶); Lu *et al.* (2002 ▶). For background to Betti-type reactions, see: Pu & Yu (2001 ▶).
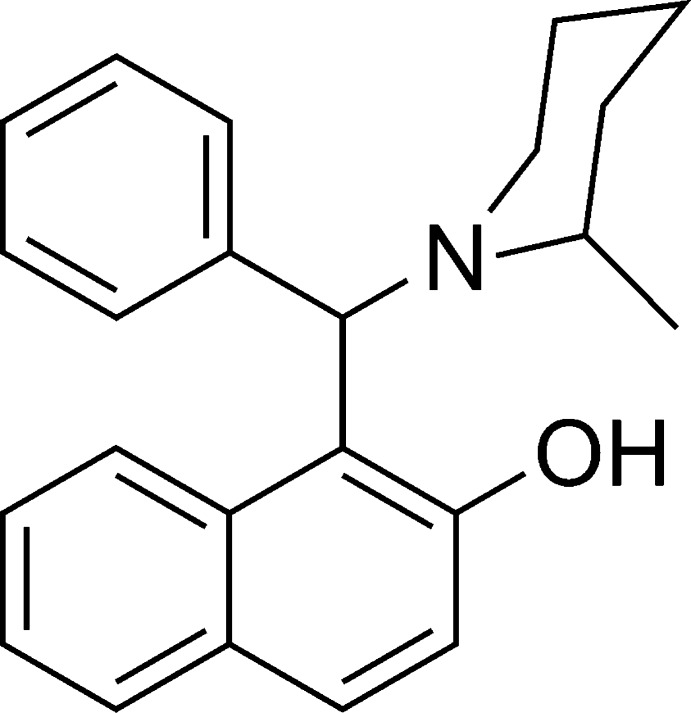


## Experimental

### 

#### Crystal data

C_23_H_25_NO
*M* *_r_* = 331.44
Orthorhombic, 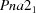

*a* = 10.249 (2) Å
*b* = 13.182 (3) Å
*c* = 13.435 (3) Å
*V* = 1815.1 (6) Å^3^
*Z* = 4
Mo *K*α radiation
μ = 0.07 mm^−1^
*T* = 293 K
0.34 × 0.32 × 0.26 mm

#### Data collection

Rigaku SCXmini diffractometer
Absorption correction: multi-scan (*CrystalClear*; Rigaku, 2005 ▶) *T*_min_ = 0.097, *T*_max_ = 0.099
18098 measured reflections
2170 independent reflections
1718 reflections with *I* > 2σ(*I*)
*R*_int_ = 0.058

#### Refinement

*R*[*F* ^2^ > 2σ(*F* ^2^)] = 0.054
*wR*(*F* ^2^) = 0.162
*S* = 1.13
2170 reflections
228 parameters
1 restraint
H-atom parameters constrained
Δρ_max_ = 0.32 e Å^−3^
Δρ_min_ = −0.20 e Å^−3^

### 

Data collection: *CrystalClear* (Rigaku, 2005 ▶); cell refinement: *CrystalClear*; data reduction: *CrystalClear*; program(s) used to solve structure: *SHELXS97* (Sheldrick, 2008 ▶); program(s) used to refine structure: *SHELXL97* (Sheldrick, 2008 ▶); molecular graphics: *SHELXTL* (Sheldrick, 2008 ▶); software used to prepare material for publication: *SHELXTL*.

## Supplementary Material

Crystal structure: contains datablock(s) I, global. DOI: 10.1107/S1600536812038652/cv5314sup1.cif

Structure factors: contains datablock(s) I. DOI: 10.1107/S1600536812038652/cv5314Isup2.hkl

Supplementary material file. DOI: 10.1107/S1600536812038652/cv5314Isup3.cml

Additional supplementary materials:  crystallographic information; 3D view; checkCIF report

## Figures and Tables

**Table 1:** Hydrogen-bond geometry (Å, °)

*D*—H⋯*A*	*D*—H	H⋯*A*	*D*⋯*A*	*D*—H⋯*A*
O1—H1*A*⋯N1	0.82	1.85	2.581 (4)	148

**Fig. 1.**
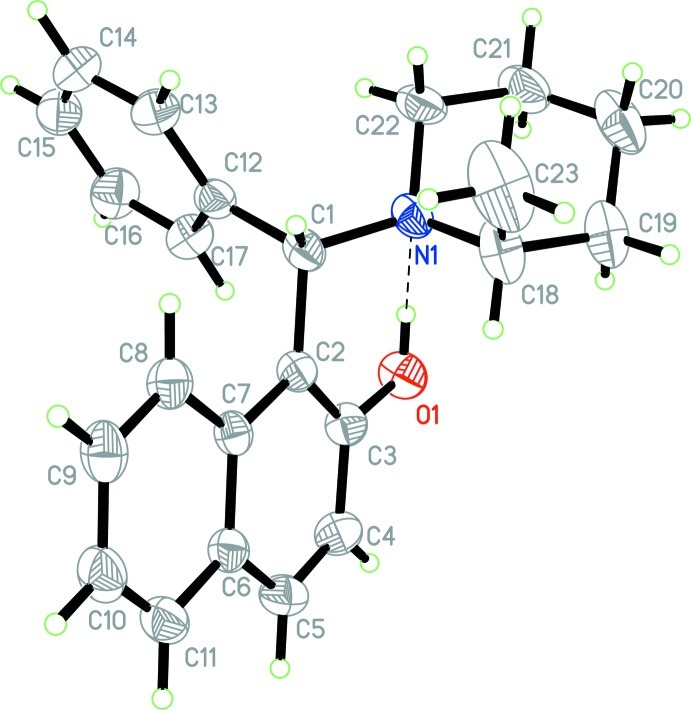
The molecular structure of the title compound with displacement ellipsoids drawn at the 30% probability level. Intramolecular hydrogen bond is shown as dashed line.

## supplementary crystallographic information

### Comment

The so-called Betti base derivatives, which can be synthesized by many ways (Pu & Yu, 2001), are important for coordination chemistry. Herein we present the title compound, (I), obtained by solvent free, one-pot, three-component domino reaction of naphthalen-2-ol, benzaldehyde and 2-methylpiperidine.

In (I) (Fig. 1), the bond lengths and angles are within the expected ranges corresponding to those observed in the related compounds (Wang & Zhao 2009; Lu *et al.* 2002). The dihedral angle between the naphthalene ring system and the benzene ring is 75.8 (2)°. The piperidine ring adopts a chair conformation, An intramolecular O—H···N hydrogen bond (Table 1) stabilize the molecular conformation. The crystal packing exhibits no short intermolecular contacts.

### Experimental

A dry 50 ml flask was charged with benzaldehyde (10 mmol), naphthalen-2-ol (10 mmol) and 2-methylpiperidine (10 mmol). The mixture was stirred at 100°C for 5 h and then ethanol (15 ml) was added. After refluxing for 30 minutes, the mixed solution was filtered and crystals of the title compound suitable for X-ray analysis was obtained by slow evaporation.

### Refinement

All H atoms were geometrically positioned and refined using a riding model, with C—H = 0.93–0.98 Å, O—H = 0.82 Å, and with *U*_iso_(H) = 1.2 *U*_eq_(C) or 1.5 *U*_eq_(C, O) for methyl and hydroxy H atoms.

In the absence of any significant anomalous scatterers in the molecule, the 1975 Friedel pairs were merged before the final refinement.

### Crystal data

**Table:**

C_23_H_25_NO	*F*(000) = 712
*M**_r_* = 331.44	*D*_x_ = 1.213 Mg m^−^^3^
Orthorhombic, *P**n**a*2_1_	Mo *K*α radiation, λ = 0.71073 Å
Hall symbol: P 2c -2n	Cell parameters from 2170 reflections
*a* = 10.249 (2) Å	θ = 2.2–27.5°
*b* = 13.182 (3) Å	µ = 0.07 mm^−^^1^
*c* = 13.435 (3) Å	*T* = 293 K
*V* = 1815.1 (6) Å^3^	Block, pale yellow
*Z* = 4	0.34 × 0.32 × 0.26 mm

### Data collection

**Table:**

Rigaku SCXmini diffractometer	2170 independent reflections
Radiation source: fine-focus sealed tube	1718 reflections with *I* > 2σ(*I*)
Graphite monochromator	*R*_int_ = 0.058
Detector resolution: 13.6612 pixels mm^-1^	θ_max_ = 27.5°, θ_min_ = 2.2°
CCD_Profile_fitting scans	*h* = −13→13
Absorption correction: multi-scan (*CrystalClear*; Rigaku, 2005)	*k* = −17→17
*T*_min_ = 0.097, *T*_max_ = 0.099	*l* = −17→17
18098 measured reflections	

### Refinement

**Table:**

Refinement on *F*^2^	Primary atom site location: structure-invariant direct methods
Least-squares matrix: full	Secondary atom site location: difference Fourier map
*R*[*F*^2^ > 2σ(*F*^2^)] = 0.054	Hydrogen site location: inferred from neighbouring sites
*wR*(*F*^2^) = 0.162	H-atom parameters constrained
*S* = 1.13	*w* = 1/[σ^2^(*F*_o_^2^) + (0.0945*P*)^2^ + 0.0807*P*] where *P* = (*F*_o_^2^ + 2*F*_c_^2^)/3
2170 reflections	(Δ/σ)_max_ < 0.001
228 parameters	Δρ_max_ = 0.32 e Å^−^^3^
1 restraint	Δρ_min_ = −0.20 e Å^−^^3^

### Special details

**Table:**

Geometry. All e.s.d.'s (except the e.s.d. in the dihedral angle between two l.s. planes) are estimated using the full covariance matrix. The cell e.s.d.'s are taken into account individually in the estimation of e.s.d.'s in distances, angles and torsion angles; correlations between e.s.d.'s in cell parameters are only used when they are defined by crystal symmetry. An approximate (isotropic) treatment of cell e.s.d.'s is used for estimating e.s.d.'s involving l.s. planes.
Refinement. Refinement of *F*^2^ against ALL reflections. The weighted *R*-factor *wR* and goodness of fit *S* are based on *F*^2^, conventional *R*-factors *R* are based on *F*, with *F* set to zero for negative *F*^2^. The threshold expression of *F*^2^ > σ(*F*^2^) is used only for calculating *R*-factors(gt) *etc*. and is not relevant to the choice of reflections for refinement. *R*-factors based on *F*^2^ are statistically about twice as large as those based on *F*, and *R*- factors based on ALL data will be even larger.

### Fractional atomic coordinates and isotropic or equivalent isotropic displacement parameters (Å^2^)

**Table:**

	*x*	*y*	*z*	*U*_iso_*/*U*_eq_	
C1	0.6335 (3)	0.5998 (2)	0.4126 (3)	0.0416 (7)	
H1	0.5935	0.6338	0.4698	0.050*	
C2	0.7644 (3)	0.5578 (2)	0.4460 (2)	0.0385 (7)	
C3	0.8185 (3)	0.4734 (2)	0.4004 (2)	0.0419 (7)	
C4	0.9413 (4)	0.4362 (3)	0.4301 (3)	0.0506 (9)	
H4	0.9770	0.3805	0.3975	0.061*	
C5	1.0082 (4)	0.4805 (3)	0.5052 (3)	0.0530 (9)	
H5	1.0886	0.4541	0.5243	0.064*	
C6	0.9578 (3)	0.5657 (3)	0.5550 (2)	0.0432 (8)	
C7	0.8359 (3)	0.6068 (2)	0.5242 (2)	0.0377 (7)	
C8	0.7907 (4)	0.6945 (3)	0.5741 (3)	0.0471 (8)	
H8	0.7121	0.7239	0.5549	0.057*	
C9	0.8611 (4)	0.7370 (3)	0.6504 (3)	0.0570 (10)	
H9	0.8293	0.7945	0.6824	0.068*	
C10	0.9793 (4)	0.6952 (3)	0.6805 (3)	0.0597 (10)	
H10	1.0260	0.7246	0.7324	0.072*	
C11	1.0262 (4)	0.6118 (3)	0.6343 (3)	0.0564 (10)	
H11	1.1051	0.5842	0.6552	0.068*	
C12	0.6500 (3)	0.6787 (2)	0.3296 (3)	0.0440 (8)	
C13	0.5835 (4)	0.7694 (3)	0.3331 (4)	0.0680 (12)	
H13	0.5275	0.7824	0.3860	0.082*	
C14	0.5983 (5)	0.8419 (3)	0.2589 (5)	0.0799 (14)	
H14	0.5513	0.9022	0.2620	0.096*	
C15	0.6808 (5)	0.8253 (3)	0.1821 (4)	0.0728 (13)	
H15	0.6912	0.8744	0.1330	0.087*	
C16	0.7491 (5)	0.7361 (4)	0.1767 (4)	0.0680 (12)	
H16	0.8050	0.7242	0.1235	0.082*	
C17	0.7347 (4)	0.6639 (3)	0.2505 (3)	0.0535 (9)	
H17	0.7828	0.6042	0.2470	0.064*	
C18	0.4314 (4)	0.5414 (3)	0.3252 (4)	0.0650 (12)	
H18A	0.4571	0.5831	0.2691	0.078*	
H18B	0.3718	0.5803	0.3662	0.078*	
C19	0.3633 (5)	0.4449 (3)	0.2877 (5)	0.0781 (15)	
H19A	0.2838	0.4635	0.2531	0.094*	
H19B	0.4199	0.4105	0.2407	0.094*	
C20	0.3305 (5)	0.3737 (4)	0.3718 (4)	0.0833 (16)	
H20A	0.2629	0.4035	0.4130	0.100*	
H20B	0.2974	0.3105	0.3449	0.100*	
C21	0.4491 (5)	0.3531 (4)	0.4338 (4)	0.0765 (14)	
H21A	0.5118	0.3152	0.3945	0.092*	
H21B	0.4246	0.3115	0.4904	0.092*	
C22	0.5127 (5)	0.4498 (4)	0.4711 (3)	0.0703 (13)	
H22	0.5941	0.4312	0.5047	0.084*	
C23	0.4262 (8)	0.5059 (6)	0.5467 (5)	0.120 (3)	
H23A	0.3499	0.5315	0.5137	0.180*	
H23B	0.4741	0.5613	0.5752	0.180*	
H23C	0.4005	0.4598	0.5985	0.180*	
N1	0.5467 (3)	0.5133 (2)	0.3834 (2)	0.0442 (7)	
O1	0.7570 (3)	0.4224 (2)	0.3269 (2)	0.0563 (7)	
H1A	0.6793	0.4373	0.3270	0.084*	

### Atomic displacement parameters (Å^2^)

**Table:**

	*U*^11^	*U*^22^	*U*^33^	*U*^12^	*U*^13^	*U*^23^
C1	0.0343 (16)	0.0435 (17)	0.0469 (18)	−0.0004 (12)	0.0041 (13)	−0.0160 (14)
C2	0.0350 (16)	0.0405 (16)	0.0401 (17)	−0.0022 (12)	0.0054 (13)	−0.0026 (13)
C3	0.0427 (17)	0.0414 (16)	0.0415 (18)	0.0026 (13)	0.0056 (14)	−0.0064 (14)
C4	0.049 (2)	0.0487 (19)	0.054 (2)	0.0105 (15)	0.0051 (16)	−0.0041 (16)
C5	0.0392 (18)	0.061 (2)	0.059 (2)	0.0085 (16)	0.0010 (16)	0.0044 (18)
C6	0.0407 (17)	0.0453 (18)	0.0435 (18)	−0.0080 (13)	0.0016 (14)	0.0085 (13)
C7	0.0391 (16)	0.0371 (14)	0.0368 (15)	−0.0072 (12)	0.0023 (13)	0.0022 (12)
C8	0.048 (2)	0.0457 (19)	0.0472 (19)	−0.0036 (14)	0.0029 (16)	−0.0080 (15)
C9	0.072 (3)	0.0490 (19)	0.050 (2)	−0.0160 (18)	0.0031 (19)	−0.0113 (17)
C10	0.073 (3)	0.059 (2)	0.047 (2)	−0.0227 (19)	−0.019 (2)	−0.0014 (18)
C11	0.054 (2)	0.063 (2)	0.052 (2)	−0.0093 (18)	−0.0151 (18)	0.0111 (18)
C12	0.0361 (17)	0.0417 (16)	0.0543 (19)	−0.0002 (12)	−0.0070 (15)	−0.0090 (15)
C13	0.053 (2)	0.055 (2)	0.095 (3)	0.0091 (18)	0.003 (2)	−0.003 (2)
C14	0.067 (3)	0.049 (2)	0.123 (4)	0.009 (2)	−0.012 (3)	0.015 (3)
C15	0.071 (3)	0.063 (3)	0.085 (3)	−0.009 (2)	−0.029 (3)	0.020 (2)
C16	0.079 (3)	0.072 (3)	0.053 (2)	−0.002 (2)	−0.002 (2)	0.008 (2)
C17	0.062 (2)	0.049 (2)	0.049 (2)	0.0051 (16)	−0.0020 (17)	−0.0045 (16)
C18	0.040 (2)	0.056 (2)	0.099 (3)	0.0055 (16)	−0.021 (2)	−0.028 (2)
C19	0.054 (3)	0.066 (3)	0.114 (4)	−0.004 (2)	−0.023 (3)	−0.030 (3)
C20	0.060 (3)	0.077 (3)	0.114 (4)	−0.027 (2)	0.029 (3)	−0.038 (3)
C21	0.082 (3)	0.071 (3)	0.076 (3)	−0.031 (2)	0.021 (3)	−0.013 (2)
C22	0.080 (3)	0.073 (3)	0.057 (2)	−0.034 (2)	0.014 (2)	−0.007 (2)
C23	0.135 (6)	0.143 (6)	0.082 (4)	−0.052 (5)	0.034 (4)	−0.037 (4)
N1	0.0385 (14)	0.0450 (15)	0.0491 (16)	−0.0056 (11)	0.0005 (12)	−0.0121 (12)
O1	0.0545 (16)	0.0564 (15)	0.0581 (16)	0.0022 (11)	−0.0002 (13)	−0.0246 (13)

### Geometric parameters (Å, º)

**Table:**

C1—N1	1.498 (4)	C14—H14	0.9300
C1—C2	1.519 (5)	C15—C16	1.370 (7)
C1—C12	1.534 (5)	C15—H15	0.9300
C1—H1	0.9800	C16—C17	1.382 (6)
C2—C3	1.387 (4)	C16—H16	0.9300
C2—C7	1.434 (5)	C17—H17	0.9300
C3—O1	1.350 (4)	C18—N1	1.464 (5)
C3—C4	1.408 (5)	C18—C19	1.535 (5)
C4—C5	1.353 (6)	C18—H18A	0.9700
C4—H4	0.9300	C18—H18B	0.9700
C5—C6	1.406 (5)	C19—C20	1.506 (8)
C5—H5	0.9300	C19—H19A	0.9700
C6—C11	1.414 (5)	C19—H19B	0.9700
C6—C7	1.422 (5)	C20—C21	1.499 (8)
C7—C8	1.415 (5)	C20—H20A	0.9700
C8—C9	1.373 (5)	C20—H20B	0.9700
C8—H8	0.9300	C21—C22	1.517 (6)
C9—C10	1.392 (6)	C21—H21A	0.9700
C9—H9	0.9300	C21—H21B	0.9700
C10—C11	1.350 (6)	C22—N1	1.486 (5)
C10—H10	0.9300	C22—C23	1.538 (8)
C11—H11	0.9300	C22—H22	0.9800
C12—C13	1.378 (5)	C23—H23A	0.9600
C12—C17	1.387 (5)	C23—H23B	0.9600
C13—C14	1.389 (8)	C23—H23C	0.9600
C13—H13	0.9300	O1—H1A	0.8200
C14—C15	1.351 (8)		
			
N1—C1—C2	109.0 (3)	C16—C15—H15	120.0
N1—C1—C12	113.0 (3)	C15—C16—C17	119.9 (5)
C2—C1—C12	111.4 (3)	C15—C16—H16	120.0
N1—C1—H1	107.7	C17—C16—H16	120.0
C2—C1—H1	107.7	C16—C17—C12	121.3 (4)
C12—C1—H1	107.7	C16—C17—H17	119.3
C3—C2—C7	118.7 (3)	C12—C17—H17	119.3
C3—C2—C1	120.9 (3)	N1—C18—C19	109.4 (3)
C7—C2—C1	120.3 (3)	N1—C18—H18A	109.8
O1—C3—C2	122.4 (3)	C19—C18—H18A	109.8
O1—C3—C4	116.8 (3)	N1—C18—H18B	109.8
C2—C3—C4	120.8 (3)	C19—C18—H18B	109.8
C5—C4—C3	121.0 (3)	H18A—C18—H18B	108.2
C5—C4—H4	119.5	C20—C19—C18	111.8 (4)
C3—C4—H4	119.5	C20—C19—H19A	109.2
C4—C5—C6	120.9 (3)	C18—C19—H19A	109.2
C4—C5—H5	119.6	C20—C19—H19B	109.2
C6—C5—H5	119.6	C18—C19—H19B	109.2
C5—C6—C11	121.3 (3)	H19A—C19—H19B	107.9
C5—C6—C7	119.3 (3)	C21—C20—C19	110.4 (4)
C11—C6—C7	119.4 (3)	C21—C20—H20A	109.6
C8—C7—C6	117.5 (3)	C19—C20—H20A	109.6
C8—C7—C2	123.1 (3)	C21—C20—H20B	109.6
C6—C7—C2	119.4 (3)	C19—C20—H20B	109.6
C9—C8—C7	121.0 (4)	H20A—C20—H20B	108.1
C9—C8—H8	119.5	C20—C21—C22	112.3 (4)
C7—C8—H8	119.5	C20—C21—H21A	109.1
C8—C9—C10	120.9 (4)	C22—C21—H21A	109.1
C8—C9—H9	119.6	C20—C21—H21B	109.1
C10—C9—H9	119.6	C22—C21—H21B	109.1
C11—C10—C9	119.9 (4)	H21A—C21—H21B	107.9
C11—C10—H10	120.0	N1—C22—C21	108.2 (3)
C9—C10—H10	120.0	N1—C22—C23	112.8 (5)
C10—C11—C6	121.3 (4)	C21—C22—C23	112.0 (4)
C10—C11—H11	119.3	N1—C22—H22	107.9
C6—C11—H11	119.3	C21—C22—H22	107.9
C13—C12—C17	117.2 (4)	C23—C22—H22	107.9
C13—C12—C1	120.7 (4)	C22—C23—H23A	109.5
C17—C12—C1	122.1 (3)	C22—C23—H23B	109.5
C12—C13—C14	121.3 (5)	H23A—C23—H23B	109.5
C12—C13—H13	119.4	C22—C23—H23C	109.5
C14—C13—H13	119.4	H23A—C23—H23C	109.5
C15—C14—C13	120.3 (4)	H23B—C23—H23C	109.5
C15—C14—H14	119.8	C18—N1—C22	112.1 (3)
C13—C14—H14	119.8	C18—N1—C1	115.3 (3)
C14—C15—C16	119.9 (4)	C22—N1—C1	111.1 (3)
C14—C15—H15	120.0	C3—O1—H1A	109.5
			
N1—C1—C2—C3	36.0 (4)	N1—C1—C12—C13	102.8 (4)
C12—C1—C2—C3	−89.4 (3)	C2—C1—C12—C13	−134.1 (4)
N1—C1—C2—C7	−145.5 (3)	N1—C1—C12—C17	−79.4 (4)
C12—C1—C2—C7	89.1 (3)	C2—C1—C12—C17	43.7 (4)
C7—C2—C3—O1	179.4 (3)	C17—C12—C13—C14	1.6 (6)
C1—C2—C3—O1	−2.1 (5)	C1—C12—C13—C14	179.4 (4)
C7—C2—C3—C4	0.0 (5)	C12—C13—C14—C15	−1.1 (8)
C1—C2—C3—C4	178.5 (3)	C13—C14—C15—C16	0.7 (7)
O1—C3—C4—C5	−177.9 (4)	C14—C15—C16—C17	−0.8 (7)
C2—C3—C4—C5	1.5 (5)	C15—C16—C17—C12	1.4 (6)
C3—C4—C5—C6	−0.9 (6)	C13—C12—C17—C16	−1.7 (6)
C4—C5—C6—C11	179.3 (4)	C1—C12—C17—C16	−179.5 (4)
C4—C5—C6—C7	−1.2 (5)	N1—C18—C19—C20	55.1 (5)
C5—C6—C7—C8	−178.0 (3)	C18—C19—C20—C21	−52.4 (5)
C11—C6—C7—C8	1.5 (5)	C19—C20—C21—C22	54.4 (5)
C5—C6—C7—C2	2.6 (4)	C20—C21—C22—N1	−57.5 (5)
C11—C6—C7—C2	−177.8 (3)	C20—C21—C22—C23	67.4 (6)
C3—C2—C7—C8	178.7 (3)	C19—C18—N1—C22	−59.9 (5)
C1—C2—C7—C8	0.1 (5)	C19—C18—N1—C1	171.6 (4)
C3—C2—C7—C6	−2.0 (4)	C21—C22—N1—C18	60.9 (5)
C1—C2—C7—C6	179.5 (3)	C23—C22—N1—C18	−63.6 (5)
C6—C7—C8—C9	−1.1 (5)	C21—C22—N1—C1	−168.5 (4)
C2—C7—C8—C9	178.2 (3)	C23—C22—N1—C1	67.0 (5)
C7—C8—C9—C10	0.3 (6)	C2—C1—N1—C18	−164.4 (3)
C8—C9—C10—C11	0.1 (6)	C12—C1—N1—C18	−40.0 (4)
C9—C10—C11—C6	0.3 (6)	C2—C1—N1—C22	66.6 (4)
C5—C6—C11—C10	178.4 (4)	C12—C1—N1—C22	−168.9 (3)
C7—C6—C11—C10	−1.1 (5)		

### Hydrogen-bond geometry (Å, º)

**Table:**

*D*—H···*A*	*D*—H	H···*A*	*D*···*A*	*D*—H···*A*
O1—H1*A*···N1	0.82	1.85	2.581 (4)	148
